# AI-powered contrast-free cardiovascular magnetic resonance imaging for myocardial infarction

**DOI:** 10.3389/fcvm.2024.1457498

**Published:** 2024-11-21

**Authors:** Vedat Cicek, Ulas Bagci

**Affiliations:** Machine & Hybrid Intelligence Lab, Department of Radiology, Northwestern University, Chicago, IL, United States

**Keywords:** cardiac magnetic resonance imaging (CMR), deep learning, artificial intelligence, machine learning, contrast free, late gadolinium contrast agent enhancement

## Abstract

Cardiovascular magnetic (CMR) resonance is a versatile tool for diagnosing cardiovascular diseases. While gadolinium-based contrast agents are the gold standard for identifying myocardial infarction (MI), their use is limited in patients with allergies or impaired kidney function, affecting a significant portion of the MI population. This has led to a growing interest in developing artificial intelligence (AI)-powered CMR techniques for MI detection without contrast agents. This mini-review focuses on recent advancements in AI-powered contrast-free CMR for MI detection. We explore various AI models employed in the literature and delve into their strengths and limitations, paving the way for a comprehensive understanding of this evolving field.

## 1 Introduction

Cardiovascular diseases (CVDs), particularly ischemic heart disease (IHD), are the leading cause of global mortality (1). A critical consequence of IHD is myocardial infarction (MI), cell death due to prolonged ischemia, leading to impaired heart muscle function (2). MI occurs due to an acute interruption of blood flow to a specific region of the myocardium, leading to ischemia and subsequent necrosis of the affected tissue. The most common underlying cause is the rupture of atherosclerotic plaques within the coronary arteries, which initiates thrombus formation and subsequent arterial occlusion.

Cardiac magnetic resonance imaging (CMR) plays a critical role in the detection and characterization of MI, offering a non-invasive method to infarcts (3). CMR's ability to provide detailed tissue characterization through late gadolinium enhancement (LGE) allows for the precise identification of infarcted myocardium, distinguishing viable from non-viable tissue (4, 5). CMR provides superior spatial resolution and tissue contrast, making it a gold standard in diagnosing MI, evaluating scar tissue, detecting infarct size and guiding subsequent treatment strategies (6, 7). Hence, CMR has become the imaging gold standard for diagnosing MI. However, gadolinium-based contrast agents (GBCA), currently essential for CMR-based MI detection (8), pose limitations. These limitations include:

• **Safety Concerns:** GBCA use can be risky for patients with contrast allergies, chronic kidney disease or populations such as pregnants and children (9). The risks associated with the use of GBCAs include allergic and adverse physiological reactions, acute kidney injury, brain deposition, nephrogenic systemic fibrosis and environmental problems (10). GBCA is known to lead to allergic reactions. Patients with unrelated allergies have a 2- to 3-fold increased risk of an allergic-like contrast reaction, while those with a prior allergic history have an approximately 5-fold increased risk (11, 12). Physiologic adverse reactions may relate to molecular properties, such as direct chemotoxicity, osmotoxicity. Cardiac arrhythmias, depressed myocardial contractility, and pulmonary edema are, potentially serious physiologic reactions to GBCA. Cardiovascular effects are more frequent and significant in patients with underlying cardiac disease. Such as severe aortic stenosis, cardiac arrhythmias or cardiomyopathies (10). Acute kidney injury (AKI) is one of the most important adverse effects of GBCA. Etiologic factors that have been suggested include renal vasoconstriction and direct tubular toxicity. Recent studies have demonstrated that GBCA related AKI increases short term and long-term mortality (13) GBCA deposits in the brain regions, particularly the globus pallidus and dentate nucleus (14). A single injection of GBCA is observed even after long-term follow-up in the cerebellar parenchyma of rats (15). Nephrogenic systemic fibrosis is a scleroderma-like illness that occurs in patients with severe renal disease and after exposure to certain GBCAs (16). Additionally, GBCA may increases environmental pollutions. The increasing use of GBCA is leading to widespread contamination of freshwater and drinking water systems. Following their excretion via urine, GBCAs enter the sewage system and are released into surface waters as they are not removed by conventional sewage treatment plants (17, 18).
• **Time Constraints:** Conventional contrast-enhanced CMR scans require lengthy scan times (35–45 min) (19). Contrast-free scans offer the potential for faster scan times.
• **Accessibility:** The need for contrast agents can add complexity and cost to CMR procedures, potentially limiting accessibility.
• **Requirement Doctors:** It is essential for a qualified doctor to be present during the administration of GBCAs to ensure patient safety and manage any complications that might arise (20, 21).

### 1.1 The promise of contrast-free CMR with AI

Driven by these limitations, there is growing interest in developing non-invasive, contrast-free CMR techniques for MI detection. These techniques hold promise for:

• **Improved Safety:** Eliminating the need for GBCA would address safety concerns for patients with contraindications.
• **Enhanced Efficiency:** Faster scan times with contrast-free CMR could improve patient experience and workflow efficiency.
• **Increased Accessibility:** Simpler procedures with contrast-free CMR could make this technology more accessible to a wider range of patients.

This mini review explores the potential of AI-powered contrast-free CMR for MI detection. We examine the various AI models employed in recent studies (summarized in Table 1) and delve into their strengths and limitations to assess the feasibility and future directions of this promising approach.

**Table 1:** Studies that explore the use of ML/DL models in conjunction with native CMR techniques to predict and detect MI without contrast agents.

First author	Publication year	Patients age gender	Model training participant\outcome	Aim (ground truth)	Used model	Model performance
Xu et al. (22)	2018		165\140	MI detection (LGE)	RNN-LSTM	Accuracy: %95 Sensitivity: %90 Specifity: %98
Xu et al. (23)	2018		165\140	MI segmentation quantification (lgeradiologist)	Spatio-temporaL NN (DSTGAN)	Clasification Accuracy: %96 Sensitivity: %92 Specifity: %98
Larozzo et al. (24)	2018		50\50	MI detection (LGE)	SVM	AUC: 0.849, Sensitivity: 92%
Zhang et al. (25)	2019	%80 57 ± 12 years	299\212	MI detection (LGE)	RNN-LSTM	AUC: 0.94
Avard et al. (26)	2022		72\52	MI detection (LGE)	SVM	AUC: 0.92
Abdulkareem et al. (27)	2022		272\108	Prediction LGE results (LGE)	SVM	Accuracy: 0.68 F1: 0.63 Precision: 0.64
Zhang et al. (28)	2022	%81 64 ± 11 years	1,687\912	MI scar detection (LGE)	Generative adversarial networks	Specifity: %100 Sensitivity: %77
Zhang et al. (29)	2022		145\43	MI detection (LGE)	Generative adversarial networks	AUC: 0.83
Amyar et al. (30)	2023	%56 54 ± 18 years	3,000\1,130	MI scar detection (LGE)	Spatio-temporal NN (ST-RAN)	AUC: 0.92 TP: 0.98 FP: 0.09

## 2 Methods

### 2.1 Conventional CMR techniques for contrast-free MI detection and deep learning

CMR techniques, such as steady-state free precession cine imaging, T1 mapping, T2 mapping, and diffusion tensor imaging (DTI) provide valuable, contrast-free insights into myocardial tissue properties and pathophysiology (31). T1 mapping, in particular, shows promise for evaluating prior MI without contrast. Quantitative analysis of T1 maps demonstrates a strong correlation with histopathological findings (32). However, the diagnostic accuracy of visual T1 map analysis suffers due to factors like non-standardized map presentation and potential confounding variables (33). T2 mapping measures the transverse relaxation time, which is sensitive to water content in tissues, making it a marker for inflammation, acute injury, or edema, such as in myocarditis or acute MI (34). DTI goes beyond standard imaging by capturing the directional diffusion of water molecules in the myocardium, providing detailed information about the orientation and integrity of myocardial fibers. This allows for the detection of microstructural changes in conditions like hypertrophic cardiomyopathy or diffuse myocardial fibrosis The ability to non-invasively assess myocardial composition with these techniques is critical, but interpreting the data is complex due to the subtle variations in relaxation times between healthy and diseased tissues (35, 36). Although several CMR techniques could be used for the diagnosis of cardiovascular diseases, LGE-MR appears to offer advantages in detecting small or subendocardial infarcts of MI with high accuracy and is well validated (37–39). LGE-MRI remains the imaging gold standard for diagnosing myocardial infarction (MI) and assessing scar tissue (40). However, GBCAs used in LGE-MRI can pose safety concerns for some patients.

Deep learning (DL), a powerful branch of machine learning, utilizes artificial neural networks to achieve high accuracy in various applications, including medical imaging reconstruction and aiding diagnostic tasks. In the context of CMR, DL holds promise for improving disease detection, diagnosis, prediction, and prognosis (41). In developing DL models for CMR analysis, key image features like contrast, noise, texture, and motion are integrated into a feature set used for training. The models optimize their parameters based on expert-labeled ground truth data. For tasks such as image segmentation, DL models extract essential data features to make accurate predictions, applicable to both classification (e.g., disease presence) and regression (e.g., extent of myocardial infarction) (42). In tasks like myocardial contouring, DL methods, particularly convolutional neural networks (CNNs), automatically learn image features for contour prediction. CNNs, composed of convolutional, pooling, fully connected, and SoftMax layers, are extensively used in image analysis (43, 44). More recently, Recurrent Neural Networks (RNNs) and Transformers have been successfully applied to extract informative features from CMR images and classify MI (45). Hybrid approaches combining multiple techniques can further enhance performance too. The main premise behind these methods to utilize ground truth labels with input images (CMR) without the need for contrast and force the neural networks to match the input data to ground truth labels.

### 2.2 Search criteria

**Figure 1:**
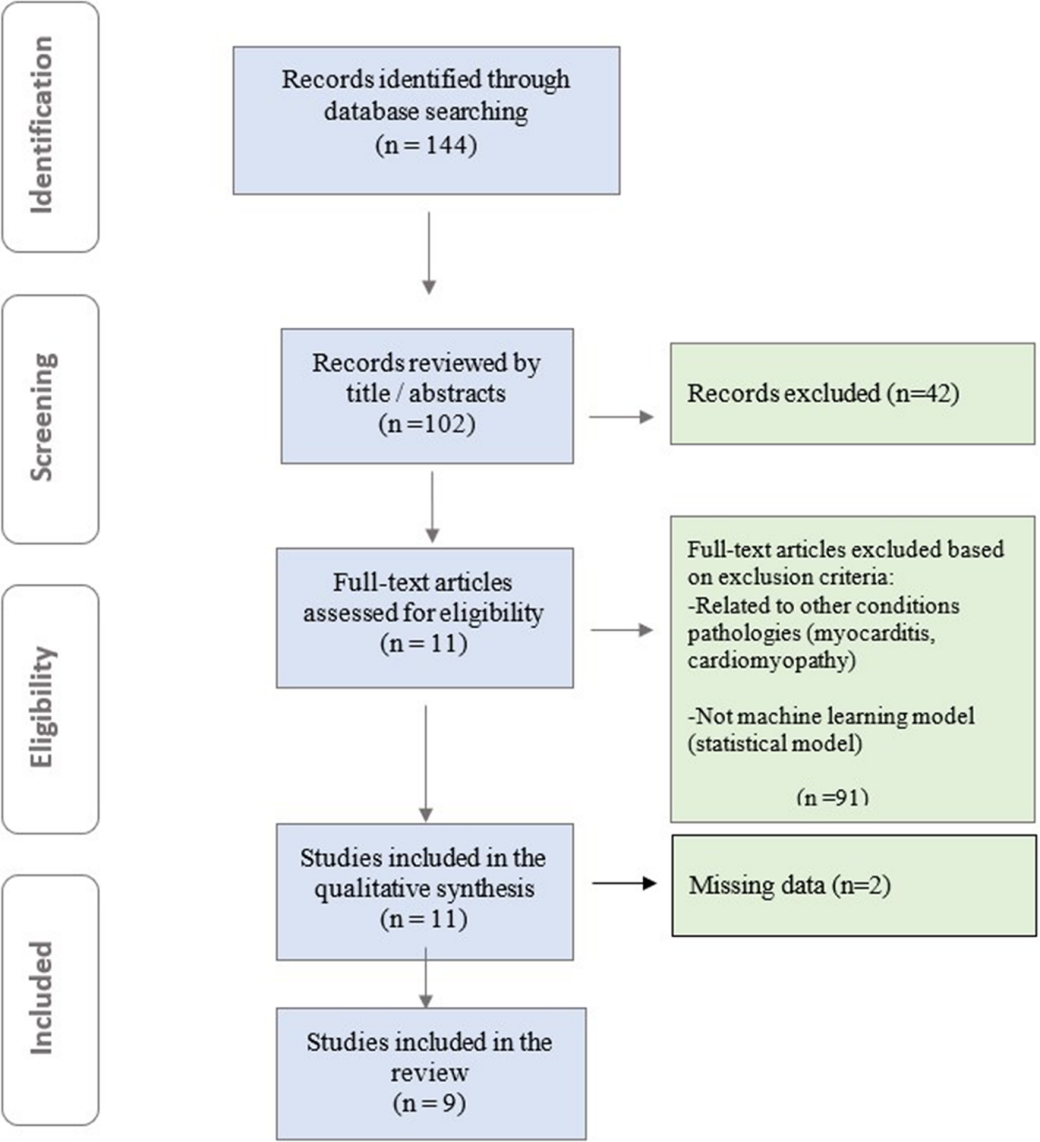
Flowchart for the study screening and selection.

To comprehensively identify relevant studies for this mini-review, we conducted a systematic search across PubMed, Embase, and Cochrane databases. Our search strategy employed a combination of Medical Subject Headings (MeSH) terms and relevant keywords, including “cardiac magnetic resonance imaging (CMR)”, “contrast-free”, “machine learning”, “artificial intelligence”, “deep learning”, “myocardial infarction”, and “coronary artery disease ”. Authors rigorously evaluated the full texts of all eligible studies using a standardized data extraction form. This form captured key study characteristics, including first author, publication year, study design (prospective/retrospective), patient population size, specific ML/DL model employed, and the area under the receiver operating characteristic curve (AUC) for the ML model compared to other models (if applicable). This structured approach ensured consistent data extraction and facilitated a comprehensive analysis of both clinical and ML aspects within the studies. Models designed with clinical outcomes other than MI and those developed using techniques other than ML were excluded. The retrieved articles were assessed based on pre-defined inclusion and exclusion criteria. High-quality studies were selected as the current review focuses on AI-based detection of myocardial infarction using non-contrast CMR. In total, 144 relevant studies were identified. After reviewing records by title/abstracts, full-text articles were assessed for eligibility, and studies meeting inclusion criteria underwent qualitative synthesis. 9 papers were ultimately included in our mini review (Figure 1).

## 3 Results and discussions

It was found that all ML-based detection models developed using non-contrast CMR statistically significantly predicted MI in studies where LGE-MRI was used as ground truths. Studies have investigated the potential of non-contrast cine-CMR images as an alternative to LGE-CMR images for assessing MI location and size without GBCA injection, employing Recurrent Neural Networks and Long Short-Term Memory (RNN-LSTM) algorithms (22). RNN-LSTM resilience to long gaps enhances memory efficiency for processing segments of CMR image sequences while incorporating temporal data. By using patch sequences instead of full image sequences, pixel-wise motion feature extraction is streamlined, reducing input dimensions and aiding in the LSTM-RNN's learning process and time series prediction Xu et al. (22) validated the performance of the RNN-LSTM model using a dataset of 165 cine CMR images for delineating MI. Their experimental results demonstrated an accuracy of 0.95. In this study experimental results showed that framework has high classification accuracy compared to the ground truth. Zhang et al. (25) introduced an RNN-LSTM model for contrast-free CMR, facilitating the confirmation, detection, and delineation of chronic MI. Study participants included 212 patients with chronic MI and 87 healthy control patients. However, the authors of the two studies conducted with RNN-LSTM noted a significant limitation: both studies were conducted in a single center and with a small number of subjects.

The Spatial-Temporal Adversarial Networks (STAN) framework aims to understand normal patterns and detect anomalies by capturing their inherent spatial-temporal characteristics without relying on optical flow mapping. Xu et al. (23) presented a contrast-free deep spatiotemporal generative adversarial network for the simultaneous delineation and measurement of myocardial infarction from CMR images. Their model achieves higher segmentation and quantification accuracy, as well as more precise quantification terms compared to existing segmentation and quantification methods. The proposed ML-based model utilized a conditional GAN approach, achieving a pixel classification accuracy of 96.98%. Amyar et al. (30) introduced a residual attention block designed to extract spatial and temporal features at various scales, thereby capturing both global and local motion characteristics to detect myocardial scar using a dataset of 3,000 patients. The model yielded results with an introduced AUC of 0.84, F1 score of 0.72, and sensitivity of 0.90. To mitigate the intricacies associated with 4D convolution, an efficient training and inference strategy based on spatiotemporal factorization (4D as 3D + time) has been employed. This method enables a reduction in model parameters by a factor of 32 while preserving high performance. The proposed layer extracts spatial and temporal features while enhancing attention on features in both directions. This enables the detection of subtle differences in left ventricle myocardial texture and cardiac motion.

Support Vector Machines (SVMs) establish margins between classes to maximize the distance between margins and classes, thus minimizing classification errors. As a binary classification method, SVMs accept labeled data from two classes and generate a model file for classifying new, unlabeled or labeled data (46). Originating from Vapnik's concept of structural risk minimization, SVMs primarily operate as two-class classifiers, systematically learning linear or nonlinear class boundaries (47). Avard et al. (26) developed an SVM-based ML model employing radiomics features to distinguish between MI tissue and viable myocardium on non-contrast cine-CMR images. The authors reported an optimal performance with an area under the curve (AUC) of 0.92 ± 0.05, an F1 Score of 0.90 ± 0.02, an accuracy of 0.85 ± 0.04, a recall of 0.92 ± 0.01, and a precision of 0.88 ± 0.04. Larroza et al. (24) trained an SVM classifier to investigate the capability of texture analysis using cine-CMR images to discriminate among infarcted nonviable, viable, and remote segments. The authors demonstrated that non-viable segments can be detected on non-contrast cine-CMR images using texture analysis, with an AUC of 0.849 and a sensitivity of 92%. Abdulkareem et al. (27) developed an SVM-based AI model to predict post-contrast information (i.e., presence, location, and/or extent of myocardial infarction scar) from non-contrast data of 272 patients with diagnoses of myocardial infarction (*n* = 108) and healthy controls (*n* = 164). They used UNet for segmentation, ResNet50 for classification. The model performance was accuracy: 0.68, F1: 0.63, precision: 0.64.

Generative models are algorithms designed to learn the underlying probability distribution of a dataset, enabling the generation of new samples that closely resemble the original data. These models find applications in data augmentation, creative content generation, and other innovative domains including medical image synthesis. Generative models manifest in various forms, each exhibiting distinct characteristics and applications. GANs have recently demonstrated remarkable success in modeling distributions, particularly low-dimensional manifolds, and generating visually appealing natural images in high-dimensional data spaces. Notably, GANs achieve state-of-the-art perceptual quality for image super-resolution tasks, scaling up to 4× upscaling for natural images sourced from datasets like ImageNet. Furthermore, GANs have been deployed for tasks such as image inpainting, style transfer, and visual manipulation, exhibiting outstanding performance compared to existing alternatives (48). Zhang et al. (28) integrated cine-CMR images and native T1-mapping to generate LGE-like images using a novel DL technique termed virtual native enhancement (VNE). This methodology employed a GAN model to enhance the imaging signal in native T1-mapping and cine images. The VNE was evaluated against LGE images using linear regression, Pearson correlation, and intraclass correlation coefficients. Additionally, a histological comparison was conducted in a porcine model of MI. VNE exhibited strong correlations with LGE in quantifying scar size (R: 0.89; intraclass correlation coefficient ICC: 0.94) and transmurally (R: 0.84; intraclass correlation coefficient ICC: 0.90). It achieved an accuracy of 84% in detecting scars with a specificity of 100% and a sensitivity of 77%. Furthermore, it demonstrated excellent visuospatial agreement with the histopathological porcine model. In another study, Zhang et al. (29) utilized a GAN-based VNE technique to generate virtual images from contrast-free CMR data, which exhibited significantly better image quality for MI detection compared to LGE images (*P* < 0.001).

### 3.1 Limitations and future prospects

Several limitations warrant consideration when interpreting the current landscape of contrast-free CMR with AI for MI detection:

1. Data Heterogeneity: CMR image variability arises from differences in scanner hardware, imaging protocols, and patient populations, leading to discrepancies in resolution and quality, complicating the generalization of DL models across clinical settings.
2. Lack of Large, Annotated Datasets: A critical challenge in developing DL algorithms for CMR analysis is the lack of large, well-annotated datasets. This scarcity limits the development and external validation of robust models across diverse patient populations.
3. Limited Generalizability: Most studies involve single centers and relatively small patient populations, hindering generalizability. Future work must focus on using more patients and heterogenous with sex and races features.
4. Interpretability: (“Black Box” Algorithms): The lack of transparency in many ML models makes it challenging to understand their decision-making processes. In other words, while these algorithms can deliver accurate predictions or classifications based on input data, the process by which they reach these decisions is not readily understandable. This circumstance also emerges as a constraint in the mentioned studies.
5. Regulatory Approval: ML models for medical imaging must undergo thorough validation and obtain regulatory approval before clinical use. These lengthy, resource-intensive processes pose significant barriers to the widespread adoption of ML models in clinical practice.
6. External Validation Gap: The field currently lacks robust external validation studies to confirm the efficacy of these models in broader clinical settings. This gap underscores potential limitations or biases in the model's generalizability and emphasizes the importance of rigorous validation across diverse datasets or settings to ensure robustness and reliability.

To bridge these gaps and propel this approach towards clinical practice, future research should prioritize:

• Multicenter Trials: Conducting studies across multiple institutions with diverse patient populations. This approach provides enhanced generalizability, reduced bias, a diverse patient population, quality assurance, efficiency, and cost-effectiveness for studies.
• Multi-Reader Validation: Incorporating assessments from multiple readers to evaluate model robustness. By involving multiple readers, multi-reader validation helps reduce the influence of individual reader biases and provides a more robust evaluation of the diagnostic accuracy of the test.
• Paired Validation Studies: Directly comparing AI-based contrast-free CMR with established diagnostic methods like LGE-MRI.

By addressing these limitations, future research can pave the way for the reliable integration of contrast-free CMR with AI into clinical decision-making for MI diagnosis.

## 4 Conclusions

This mini-review explores the promise of AI-powered contrast-free CMR for MI detection. Studies using machine learning, specifically DL models with CMR techniques, demonstrate encouraging results for MI prediction without GBCAs. These models can learn to recognize patterns in the data and identify areas needing further investigation. This evolution benefits patient comfort (eliminating the need for intravenous cannulation), enhances safety, and reduces the potentially harmful environmental consequences of excreted gadolinium. Additionally, this advancement shortens the procedure time for cardiac MRI, decreases complexity, reduces healthcare costs, and increases patient efficiency, all while maintaining high quantitative and qualitative performance. These findings underscore the ability of ML/DL models to address real-world challenges in cardiovascular medicine. Future research should aim to overcome limitations such as limited generalizability and lack of external validation through multicenter, multi-reader, and paired validation studies. By addressing these challenges, AI-powered contrast-free CMR has the potential to become a reliable and valuable tool for MI diagnosis in clinical settings.
